# RETRACTION: Analysis of the Associated Factors in Postoperative Wound Infection Following Open Reduction and Internal Fixation for Elbow Fracture

**DOI:** 10.1111/iwj.70412

**Published:** 2025-03-25

## Abstract

**RETRACTION**: CuiC. , MaL. , and QiX. , “Analysis of the Associated Factors in Postoperative Wound Infection Following Open Reduction and Internal Fixation for Elbow Fracture,” International Wound Journal 21, no. 4 (2024): e14825, 10.1111/iwj.14825.38613419 PMC11015467

The above article, published online on 13 April 2024, in Wiley Online Library (http://onlinelibrary.wiley.com/), has been retracted by agreement between the journal Editor in Chief, Professor Keith Harding; and John Wiley & Sons, Ltd. Following an investigation by the publisher, all parties have concluded that this article was accepted solely on the basis of a compromised peer review process. The editors have therefore decided to retract the article. The authors agree to the retraction and have stated that they were not involved in the compromised peer review process.

**RETRACTION**: C. Cui , L. Ma , and X. Qi , “Analysis of the Associated Factors in Postoperative Wound Infection Following Open Reduction and Internal Fixation for Elbow Fracture,” International Wound Journal 21, no. 4 (2024): e14825, 10.1111/iwj.14825.38613419 PMC11015467

The above article, published online on 13 April 2024, in Wiley Online Library (http://onlinelibrary.wiley.com/), has been retracted by agreement between the journal Editor in Chief, Professor Keith Harding; and John Wiley & Sons, Ltd. Following an investigation by the publisher, all parties have concluded that this article was accepted solely on the basis of a compromised peer review process. The editors have therefore decided to retract the article. The authors agree to the retraction and have stated that they were not involved in the compromised peer review process.

